# A multi-omics approach to investigate characteristics of gut microbiota and metabolites in hypertension and diabetic nephropathy SPF rat models

**DOI:** 10.3389/fmicb.2024.1356176

**Published:** 2024-04-29

**Authors:** Jinjing Lu, Xiaoying Gong, Chenlu Zhang, Tengfei Yang, Dongmei Pei

**Affiliations:** ^1^Department of Health Management, Shengjing Hospital of China Medical University, Shenyang, China; ^2^Department of Critical Care Unit, Shengjing Hospital of China Medical University, Shenyang, China; ^3^Department of Neurosurgery, Shengjing Hospital of China Medical University, Shenyang, China

**Keywords:** multi-omics, intestinal microbiota, metabolites, hypertension, diabetic nephropathy

## Abstract

**Background:**

Imbalance in intestinal microbiota caused by microbial species and proportions or metabolites derived from microbes are associated with hypertension, as well as diabetic nephropathy. However, the involvement of the intestinal microbiota and metabolites in hypertension and diabetic nephropathy comorbidities (HDN) remains to be elucidated.

**Methods:**

We investigated the effects of intestinal microbiota on HDN in a rat model and determined the abundance of the intestinal microbiota using 16S rRNA sequencing. Changes in fecal and serum metabolites were analyzed using ultra-high-performance liquid chromatography-mass spectrometry.

**Results:**

The results showed abundance of *Proteobacteria* and *Verrucomicrobia* was substantially higher, whereas that of *Bacteroidetes* was significant lower in the HDN group than in the sham group. *Akkermansia, Bacteroides, Blautia, Turicibacter, Lactobacillus, Romboutsia, and Fusicatenibacter* were the most abundant, and *Prevotella, Lachnospiraceae_NK4A136_group*, and *Prevotella_9* were the least abundant in the HDN group. Further analysis with bile acid metabolites in serum showed that *Blautia* was negatively correlated with taurochenodeoxycholic acid, taurocholic acid, positively correlated with cholic acid and glycocholic acid in serum.

**Conclusions:**

These findings suggest that the gut microbiota and metabolites in feces and serum substantially differed between the HDN and sham groups. The F/B ratio was higher in the HDN group than in the sham group. *Blautia* is potentially associated with HDN that correlated with differentially expressed bile acid metabolites, which might regulate the pathogenesis of HDN *via* the microorganism–gut–metabolite axis.

## 1 Introduction

Diabetes mellitus (DM) is a metabolic disorder that is characterized by chronic hyperglycemia due to an absolute or relatively insufficient amount of secreted insulin. The estimated global prevalence of diabetes among people aged 20–79-years during 2021 was ~10.5% (536.6 million people) and is expected to reach 12.2% (783.2 million people) by 2045 (Sun et al., 2022). Diabetic nephropathy (DN) is a prevalent microvascular complication of diabetes and the leading cause of end-stage renal disease (ESRD) in developed countries and developed regions of China (Ma, 2018; Johansen et al., 2021). Approximately 40% of DM patients will eventually develop DN (Chen et al., 2023).

Hypertension affect ~1.5 billion (Murray and Lopez, 2013) people worldwide. Patients with diabetes have a high prevalence of hypertension (Sabuncu et al., 2021) and those with hypertension are also at increased risk of developing diabetes (Izzo et al., 2009). Since patients with both DN and hypertension are more prone to develop macrovascular (Yen et al., 2022) and microvascular complications (Brownrigg et al., 2016) that lead to a poor prognosis, understanding the underlying mechanisms is crucial.

Changes to the intestinal microbiota caused by microbial species and proportions or metabolites derived from microbes are associated with increased susceptibility to diseases (Gentile and Weir, 2018). Intestinal microbiota disorders play important roles in DM (Wu et al., 2023), DN (Zhao et al., 2023), chronic kidney disease, ESRD (Luo et al., 2023), and in DM progression to DN and subsequent ESRD (Mao et al., 2023). However, the underlying mechanisms of how intestinal flora affects DN remain uncertain.

The intestinal microbiota plays a key role in the development of hypertension (Lucas et al., 2023). However, a disordered intestinal microbiota in DN is inconsistent with hypertension. For instance, the ratio of abundance of *Firmicutes* to *Bacteroidetes* (F/B) is significantly decreased in DN (Li et al., 2020), whereas that in the intestinal contents of patients with hypertension is increased (Yang et al., 2015). These findings suggest a complex mechanism between intestinal microbiota functions and derived metabolites in DN and comorbid hypertension.

16S rDNA amplicon sequencing technology has become an important means of studying the composition and structure of microbial communities in environmental samples. Untargeted metabolomics aims to detect as many metabolites as possible in biological samples for the purpose of discovery, reflecting the overall metabolite information to the greatest extent possible, LC-MS/MS technology is used. To delve into this aspect further, in this study, we aimed to investigate changes and crucial regulatory roles of gut microbiota and their metabolites in the DN and comorbid hypertension, by feeding rats with a high-carbohydrate high-fat diet, inducing diabetes in them by injecting streptozotocin (STZ), unilaterally ligating the renal arteries, and applying non-targeted metabolomics and 16S rRNA gene sequencing. The results showed abundance of *Proteobacteria* and *Verrucomicrobia* was substantially higher, whereas that of *Bacteroidetes* was significant lower in the HDN group than in the sham group. *Akkermansia, Bacteroides, Blautia, Turicibacter, Lactobacillus, Romboutsia, and Fusicatenibacter* were the most abundant, and *Prevotella, Lachnospiraceae_NK4A136_group*, and *Prevotella_9* were the least abundant in the HDN group. Further analysis with bile acid metabolites in resum showed that *Blautia* was negatively correlated with taurochenodeoxycholic acid, taurocholic acid, positively correlated with cholic acid and glycocholic acid in serum.

## 2 Materials and methods

### 2.1 Animals

A total of 26 six-week-old SPF Sprague-Dawley rats weighing 200 g were selected for studies (Beijing Huafukang Biotechnology Co. Ltd., Beijing, China). The rats were randomly assigned to either a group with hypertension and diabetic nephropathy (HDN) or a sham group (*n* = 14 per group). We induced HDN model by feeding the rats with a high-calorie high-carbohydrate diet for 24 weeks. The purified diets were produced by Trophic Animal Feed High-Tech Co., Ltd. (Nantong, China). The diet formulas are shown in Supplementary Table 1. The HDN group was anesthetized before undergoing unilateral renal artery ligation. We intraperitoneally injected 30 mg/kg of streptozotocin into the HDN group on postoperative day (POD) 7. The sham group was fed with a standard diet (Trophic Animal Feed High-Tech Co. Ltd, Nantong, China). This group was also anesthetized and underwent an abdominal incision, and suturing; however, the renal arteries were not clipped. The sham group was intraperitoneally injected with 0.1 mol/L sodium citrate buffer (pH 4.2) on POD 7. Fasting blood glucose (FBG) was evaluated in tail tip blood and measured using an Accu-Chek Advantage glucometer (Roche Diagnostics GmbH, Mannheim, Germany). Blood pressure (BP) was determined using a tail-cuff. Rats with FBG >16.7 mmol/L and BP >140 mmHg were considered as successful models. The Institutional Animal Care and Use Committee of Shengjing Hospital of China Medical University (Shenyang, China) approved the animal experiments (2023PS1422K).

### 2.2 Sample collection

All rats were anesthetized with isoflurane before the blood was collected from the orbital venous plexus. Serum (≥ 1.5 mL) obtained by centrifugation at 300 × g was stored at −80°C. Colon contents were collected and immediately frozen with liquid nitrogen after sampling and stored at −80°C.

### 2.3 Sequencing and analysis of 16s rDNA amplicons

The purity and concentration of extracted DNA from colon contents were detected using 2% agarose gel Electrophoresis. A library was then constructed from 1 ng/μL of DNA using TruSeq^®^ DNA PCR-Free Sample Preparation Kits (Illumina, California -San Diego, USA). The library was quantified using Qubit and Q-PCR, then sequenced using a NovaSeq 6000 System (Novogene, Sacramento, CA). Filtered sequences were clustered into operational taxonomic units (OTUs) using the UPARSE-OTU algorithm, then species annotation was analyzed using Mothur and classified using the small subunit ribosomal (SSUr) RNA database. Changes in intestinal flora were analyzed using QIIME v.1.9.1 and R v. 3.5.2 (R Foundation for Statistical Computing, Vienna, Austria).

### 2.4 Sample processing and analysis

Metabolites were extracted from thawed serum samples using 80% methanol buffer. Serum was incubated on ice for 5 min followed by centrifugation at 15000 *g*, 4°C for 20 min. The supernatants were transferred to 96-well plates, dried under nitrogen and stored at −80°C. Thawed fecal samples (100 mg) were extracted using 80% methanol, centrifuged at 15000 *g*, 4°C for 20 min, then supernatants were stored at −80°C.

### 2.5 UHPLC-MS/MS

UHPLC-MS/MS analyses were performed using a Vanquish UHPLC system (ThermoFisher, Germany) coupled with an Orbitrap Q ExactiveTMHF-X mass spectrometer (Thermo Fisher, Germany) in Novogene Co., Ltd. (Beijing, China). Samples processed previously were injected onto a Hypesil Gold column (100 × 2.1 mm, 1.9 μm) using a 17-min linear gradient at a flow rate of 0.2 mL/min. The eluents for the positive polarity mode were eluent A (0.1% FA in Water) and eluent B (Methanol).The eluents for the negative polarity mode were eluent A (5 mM ammonium acetate, pH 9.0) and eluent B (Methanol).The solvent gradient was set as follows: 2% B, 1.5 min; 2%−100% B, 3 min; 100% B, 10 min;100%−2% B, 10.1 min;2% B, 12 min. Q ExactiveTM HF-X mass spectrometer was operated in positive/negative polarity mode with spray voltage of 3.5 kV, capillary temperature of 320°C, sheath gas flow rate of 35 psi and aux gas flow rate of 10 L/min, S-lens RF level of 60, Aux gas heater temperature of 350°C.

### 2.6 Metabolite analysis

The metabolites were annotated using the Encyclopedia of Genes and Genomes (KEGG), the Human Metabolome Database (HMDB), and the Lipid Metabolites and Pathways Strategy (LIPIDMaps) (https://www.lipidmaps.org/). Principal components analysis (PCA) and partial least squares discriminant analysis (PLS-DA) proceeded using metaX^®^ (https://metaxsoft.com). Statistically significant metabolite parameters comprised fold change (FC) ≥ 2 or ≤ 0.5, variable importance in projection (VIP) > 1, *p* < 0.05, then volcano plots of metabolites were plotted based on log2 FC and –log10p. Differential metabolites were visualized using clustering heat maps. Correlations among differential metabolites were calculated using Pearson coefficients. Results with *p* < 0.05 were considered to be significantly different.

### 2.7 Statistical analysis

Continuous variables are presented as means ± standard error of mean (SEM). Between-group differences were analyzed using Student *t*-tests. Relationships between species and metabolites were analyzed using Spearman rank correlations. All data were analyzed using SPSS 21 (IBM Corp., Armonk, NY, USA) and R v. 3.5.2.

## 3 Results

### 3.1 Evaluation of HDN model

**Figure 1:**
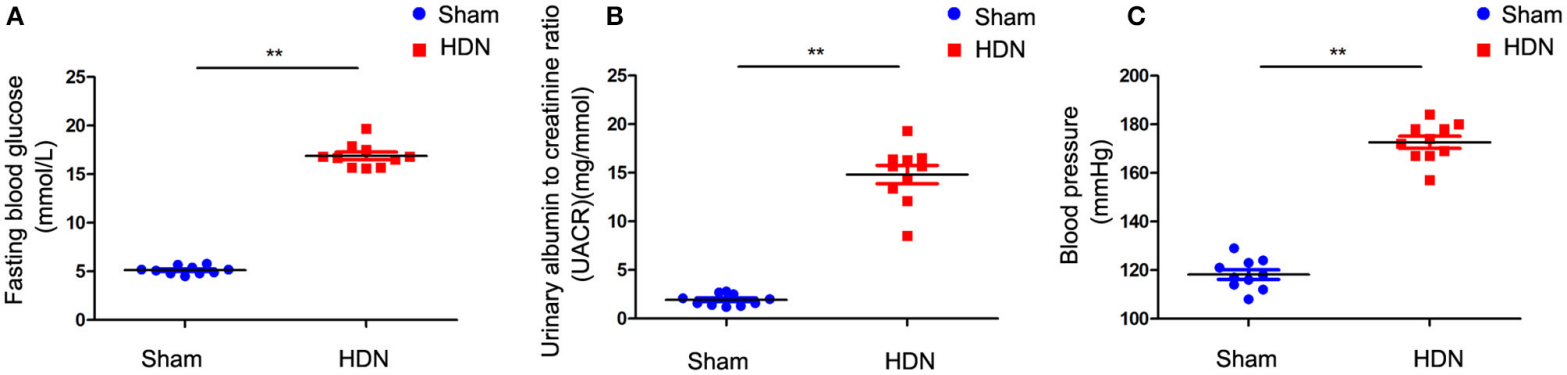
Evaluation of diabetic nephropathy-hypertension (HDN) model rats. Evaluation of **(A)** fasting blood glucose, **(B)** urinary albumin to creatinine ratios, and **(C)** blood pressure. Data are expressed as means ± SEM (*n* =10; ^**^P < 0.01).

Fasting blood glucose (FBG) levels were increased in HDN compared with sham rats (Figure 1A). The urinary albumin to creatinine ratio (UACR) was significantly increased in rats with HDN compared with sham rats (Figure 1B). These ratios are sensitive indicators for early diagnoses of renal damage in diabetes; Changes in UACR precede those of blood urea nitrogen and creatinine, indicating impaired glomerular filtration function. Moreover, BP was significantly elevated in the HDN rats (Figure 1C), confirming successful establishment of the model.

### 3.2 Gut microbial profiles

The gut microbiota notably differed between the HDN and sham rats. The quality control effective rate 81.71%, with 67,665 quality-control-validated data points, among 81,631 valid data points. We obtained 3,092 OTUs with 97% identity, then annotated them using the Silva138 database (https://www.arb-silva.de/). We annotated 1260 (40.75%) OTUs at the genus level. We identified the top-five most abundant microbiota at the phylum level using a Sankey map (Supplementary Figure 1A). Rarefaction curves indicated that the current sequencing depth adequately reflected microbial diversity (Supplementary Figures 1B, C). Rank abundance curves indicated the richness and evenness of species (Supplementary Figures 1D, E). Box plots of biodiversity and community surveys show species richness (Supplementary Figure 1F).

**Figure 2:**
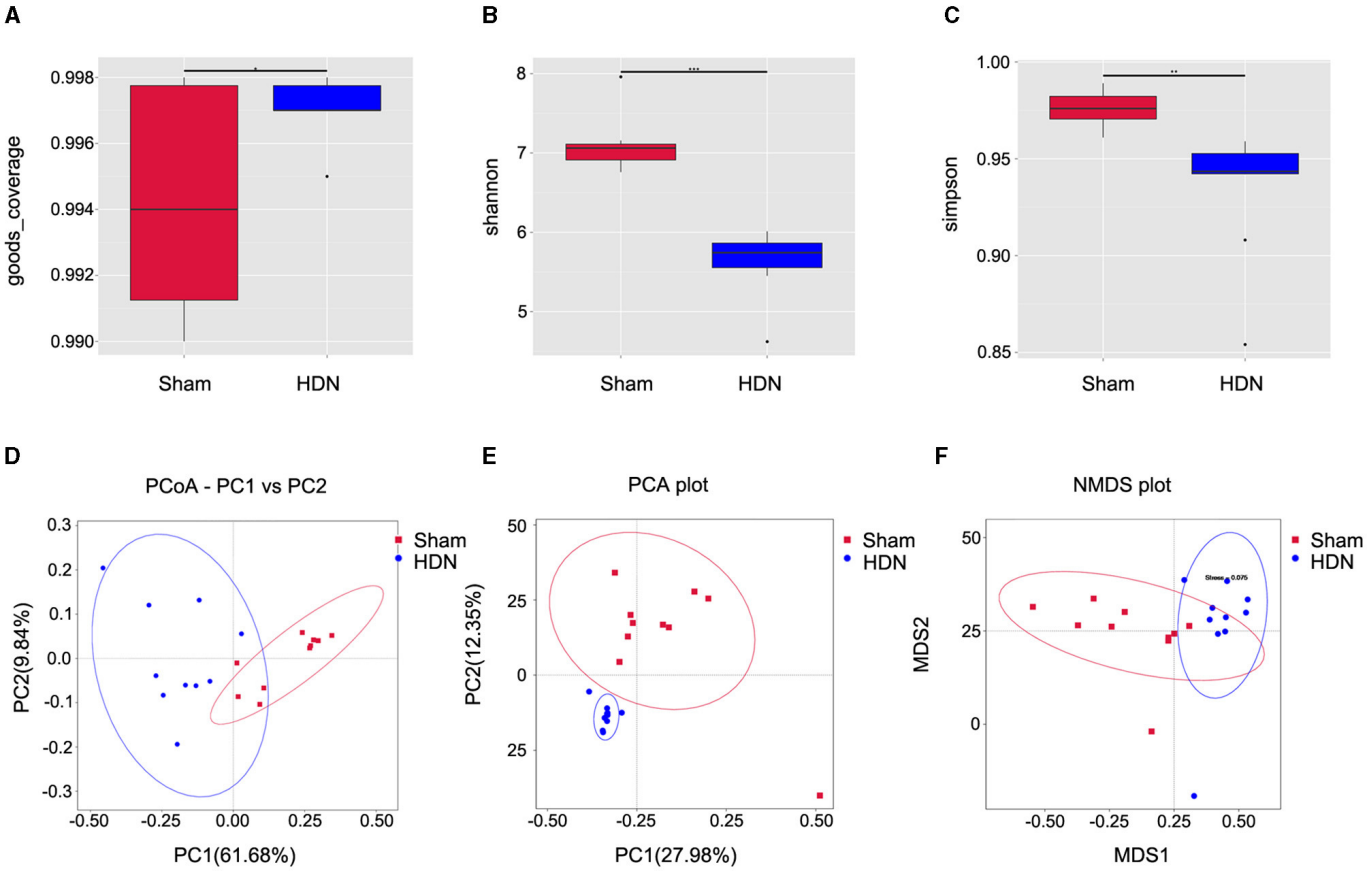
Relative bacterial richness and evenness in two groups of samples. **(A)** Good coverage of each sample library. Higher values represent a higher probability of sequence detection. **(B)** Shannon index estimates of microbial diversity. **(C)** Simpson index estimates of diversity. Assessment of gut microbiota using **(D)** PCoA (PC1 = 61.68%, PC2 = 9.84%), **(E)** PCA (PC1 = 27.98%, PC2 = 12.35%) and **(F)** NMDS analysis. Red: sham group; blue: HDN group. HDN, hypertension and diabetic nephropathy; NDMS, non-metric multidimensional scaling; PCA, principal component analysis; PCoA, principal coefficient analysis.

Alpha diversity analysis revealed significant differences in goods coverage, as well as the Shannon and Simpson indices between the HDN and sham groups (Figures 2A–C). Principal coordinate (PCoA) and principal component (PCA), analyses and non-metric multidimensional scaling (NMDS) for beta diversity, revealed differences between the groups and significant divergence in the composition and abundance of the gut microbiota (Figures 2D–F). Supplementary Table 1 shows the alpha diversity indexes (Shannon, Simpson, chao1, ACE, goods coverage, PD_whole_tree) of the samples (data volume selected during homogenization: cutoff = 46,210, at a consistency threshold of 97%).

### 3.3 Changes in composition of gut microflora associated with HDN

**Figure 3:**
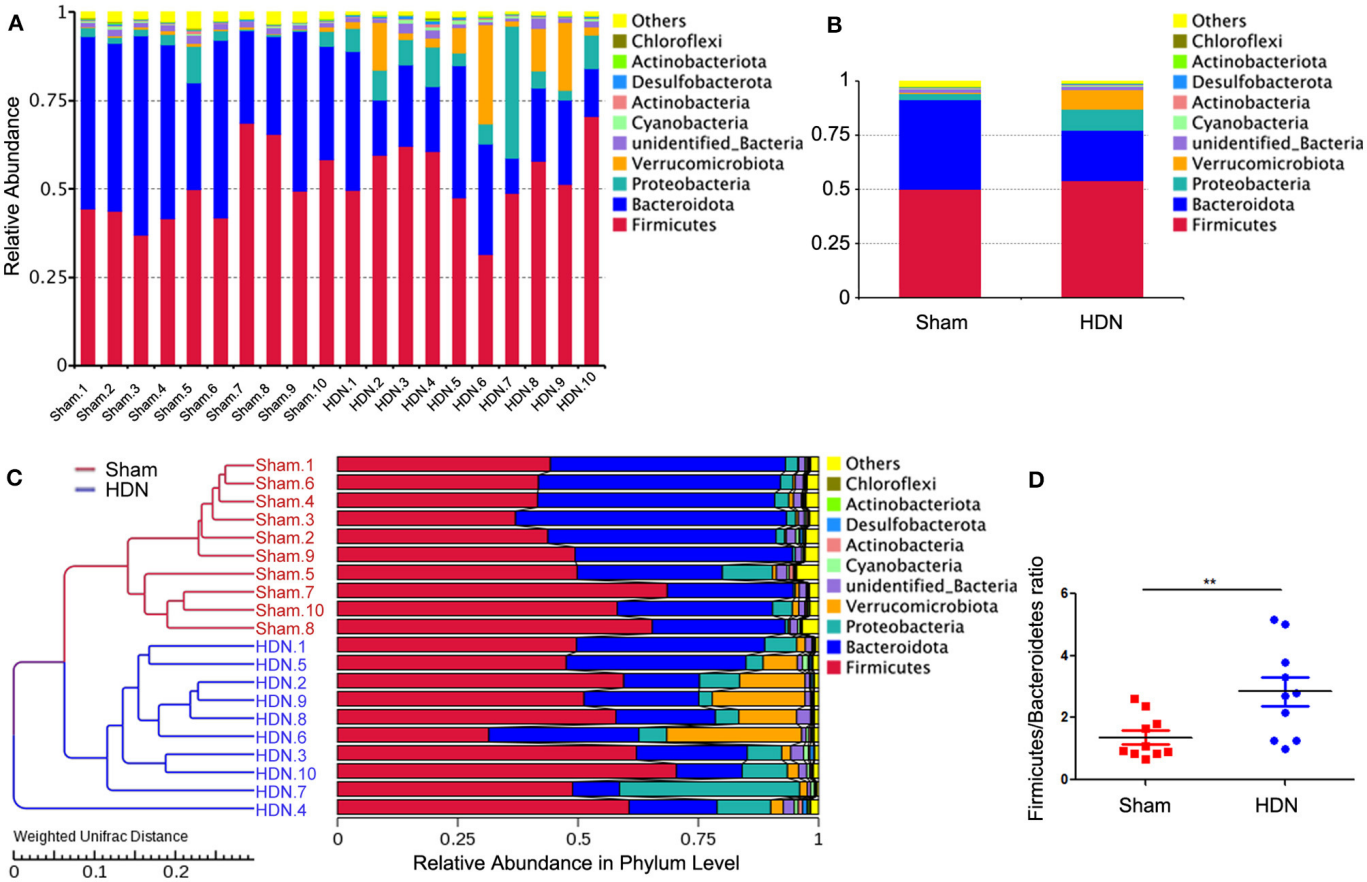
Changes in gut microflora phyla associated with HDN. Data are expressed as means ± SEM. **(A, B)** Proportions of gut microflora phyla in HDN and sham groups assessed using microbial taxa assignment. **(C)** Left, UPGMA cluster tree; right, distribution map of relative species abundance at phylum level. **(D)** Changes in *Firmicutes*/*Bacteroidetes* ratios in HDN and sham groups (*n* =10 each). **P < 0.01. HDN, hypertension and diabetic nephropathy; UPGMA, Unweighted Pair Group Method with Arithmetic Mean.

**Figure 4:**
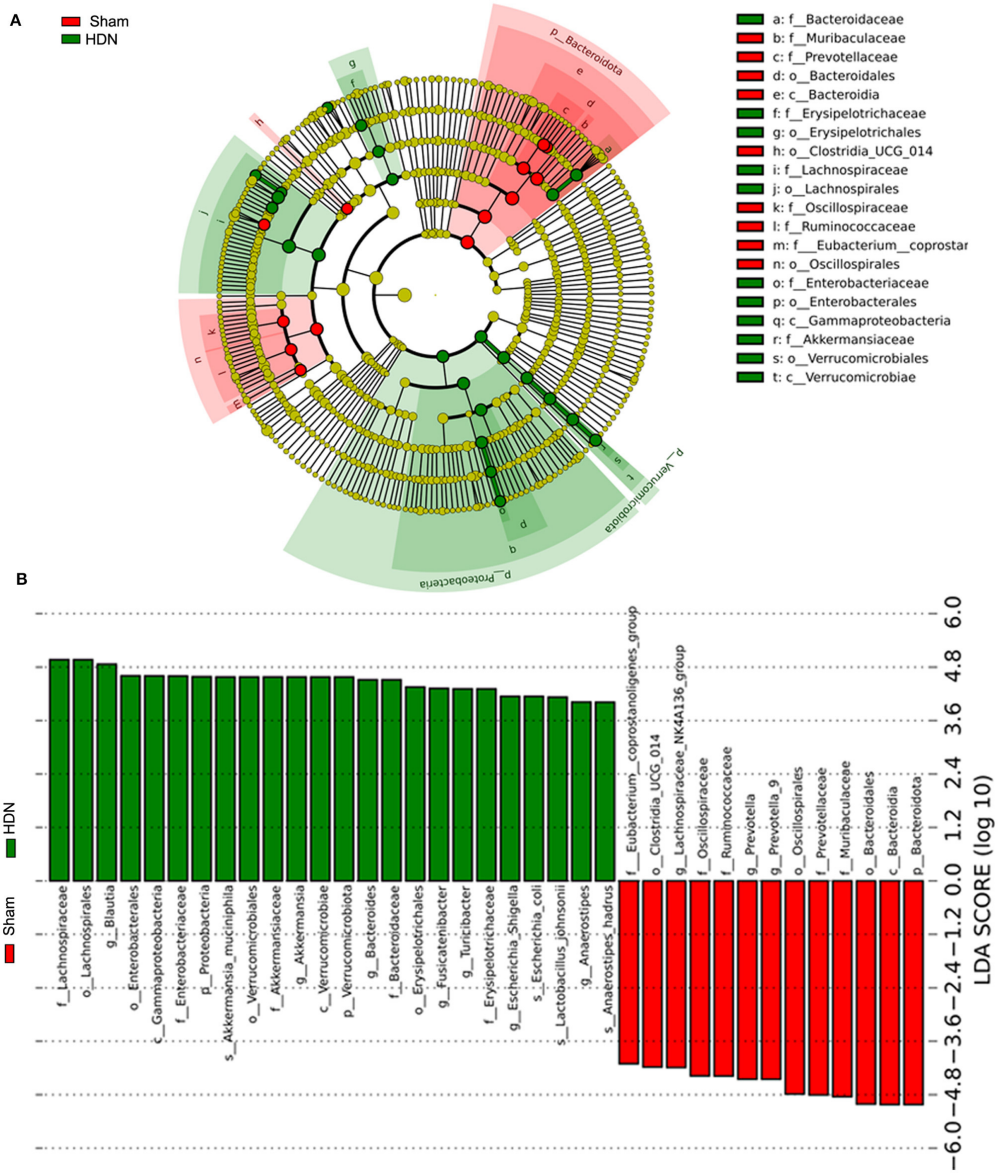
Findings of LEfSe. **(A)** Cladogram shows phylogenetic distribution of microbiota in sham and HDN groups. **(B)** Histogram of LDA scores shows effective size and rank of differentially abundant taxa. Green and red, HDN and sham groups, respectively (*n* = 10; LDA score > 4.0). HDN, hypertension and diabetic nephropathy; LDA, linear discriminant analysis; LEfSe, linear discriminant analysis effect size.

The abundance of *Proteobacteria* and *Verrucomicrobiota* increased, whereas that of *Bacteroidetes* was lower at the phylum level in the HDN group, compared with the sham group. Supplementary Figures 2A, B show that at the pylum level, *Verrucomicrobiota* was significantly increased in the HDN group, *Bacteroidetes* was decreased in the HDN group. At the genus level, *Akkermansia, Bacteroidetes* and *Blautia* were increased in the HDN group. The abundance of *Proteobacteria* was higher in the HDN group than in the sham group. The trend was similar in the HDN and sham groups for *Verrucomicrobiota*; however, *Bacteroidetes* were less abundant in the HDN group (Figures 3A, B). The abundance of *Verrucomicrobiota* (*P* = 0.0167) was significantly more, whereas *Bacteroidetes* (*P* = 0.001), *Gemmatimonadota* (*P* = 0.042), *Myxococcota* (*P* = 0.048), *Gemmatimonadetes* (*P* = 0.025), and *Elusimicrobia* (*P* = 0.004) were less abundant in the HDN group (Supplementary Table 2). Supplementary Table 3 shows that 114 genera significantly differed between the groups. The relative prevalence of species at the phylum level was determined using the unweighted pair group method with arithmetic (UPGMA) (Figure 3C). The *Firmicutes/Bacteroidetes* (F/B) ratio is associated with multiple diseases (Li et al., 2020). We found a higher F/B ratio in the HDN group (Figure 3D). We further investigated relationships among microbiota from the phylum to the genus level using linear discriminant analysis (LDA) Effect Size (LEfSe) (Figures 4A, B).

**Figure 5:**
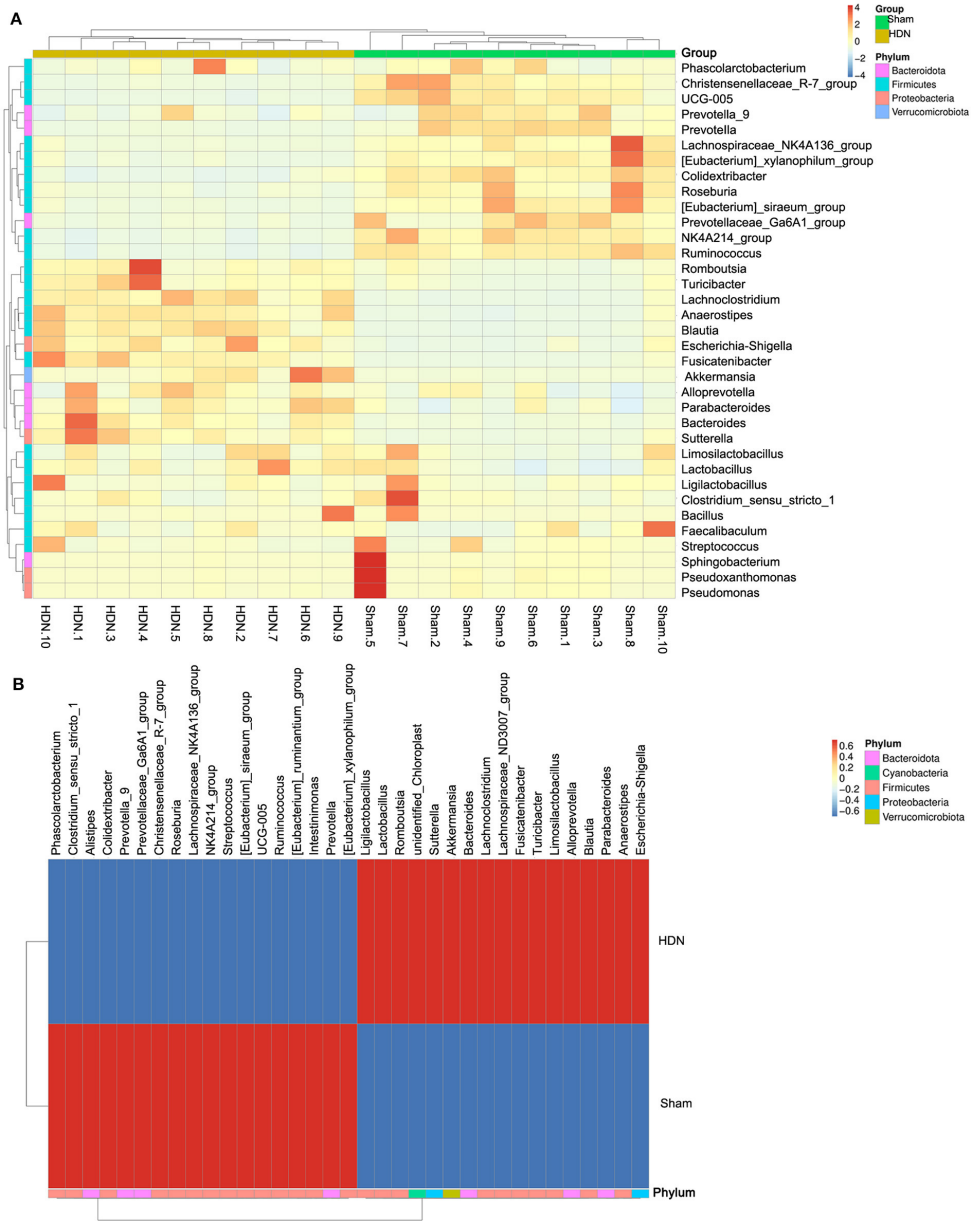
Cluster heat map of relative abundance of 35 genera. **(A)** Thirty-five OTUs are distributed at the phylum and genera levels. **(B)** Heat map shows relative abundance of differential OTUs between HDN and sham groups. Data were compared using Wilcoxon rank sum tests. Z-transformation is shown from low (blue) to high (red) abundance. HDN, hypertension and diabetic nephropathy; OTUs, operational taxonomic units.

Figure 5A shows a cluster heat map of the relative abundance of 35 genera. The overall abundance of OTUs was higher in the HDN group than in the sham group (18 vs. 17; Figure 5B). Collectively, the significant difference in abundance of microbes was sufficient to distinguish healthy from HDN rats, fed with high-carbohydrate high-fat diet.

### 3.4 Metabolomics analysis of fecal and serum samples from HDN and sham rats

The gut microbiome has effects on fecal metabolites (Wikoff et al., 2009). We investigated differences in fecal and serum metabolites between the groups using non-targeted metabolomics and LC-MS.

**Figure 6:**
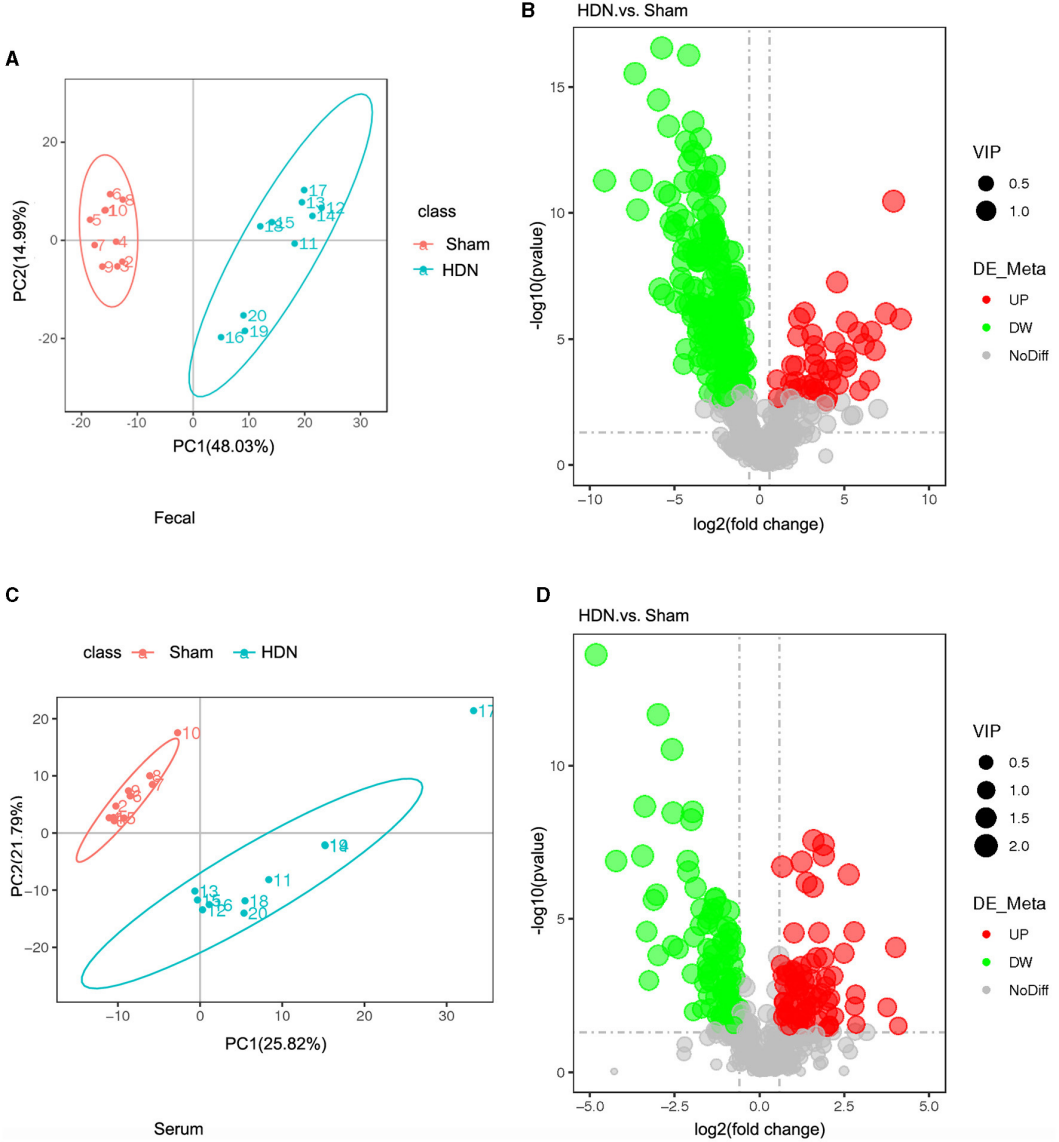
Fecal and serum metabolites differed between HDN and sham rats. **(A)** OPLS-DA scores and **(B)** volcano plot of fecal metabolites. **(C)** OPLS-DA scores and **(D)** volcano plot of serum metabolite (VIP > 1; |P_corr_ ≥ 0.5). HDN, hypertension and diabetic nephropathy; OPLS-DA, Orthogonal Projections to Latent Structures with Discriminant Analysis; VIP, variable importance in projection.

The fecal metabolites were separated in the groups (Figure 6A), indicating that HDN caused changes in fecal biomarkers. Volcano plots of 544 fecal metabolites identified by LC-MS showed that 43 and 226 of 269 fecal metabolites were significantly upregulated and downregulated, respectively (Figure 6B). Compared with the sham group, The top 10 downregulated fecal metabolites in the HDN group comprised fatty acid esters of hydroxy fatty acids (FAHFA; 18:2/20:4), acyl GlcADG (12:0-12:0-18:2), alpha-ketoglutaric acid, 2-ketohexanoic acid, 4-methylvaleric acid, dodecanedioic acid, 4-hydroxybenzoic acid, xanthosine, ursolic acid, and 3-methyladipic acid (Supplementary Table 4).

Orthogonal projections to latent structures with discriminant analysis (OPLS-DA) of serum metabolite profiles revealed good separation in the groups, suggesting that HDN altered serum biomarkers (Figure 6C). Volcano plots revealed that among 182 of 501 serum metabolites, 79 and 103 were significantly upregulated and downregulated, respectively, in the HDN group (Figure 6D). The top 10 differential downregulated metabolites in the HDN group were dihydroroseoside, equol, taurochenodeoxycholic acid (sodium salt), myricetin, taurochenodeoxycholic acid, 18-β-glycyrrhetinic acid, sulfaquinoxaline, calcitriol, 3-(2-naphthyl)-D-alanine, and taurocholic acid (Supplementary Table 5).

### 3.5 Differences between HDN and sham groups by multi-level analysis

**Figure 7:**
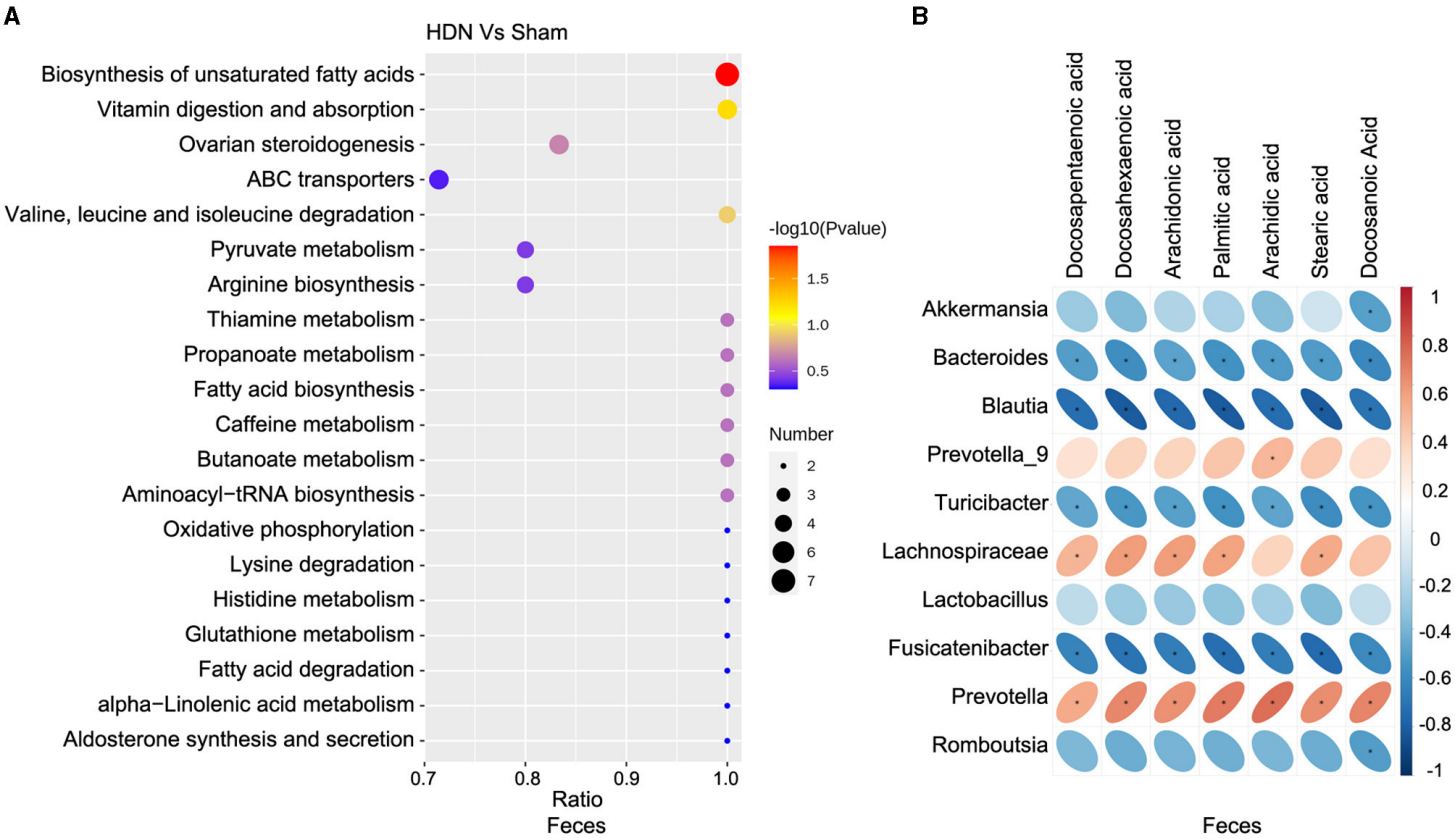
Correlations between microbiota and fecal metabolites. **(A)** Fecal metabolic changes in most relevant KEGG pathways. **(B)** Positive (red) and negative (blue) correlations between microbiota and fecal metabolites. *P < 0.05. KEGG, Kyoto Encyclopedia of Genes and Genomes.

Among differential metabolic pathways involved in HDN development identified using KEGG analysis, 69 regulated synthesis of fecal metabolites (Supplementary Table 6). Biosynthesis of unsaturated fatty acid pathways were significantly relevant to fecal metabolic alterations (Figure 7A). Among the fecal metabolites, stearic, palmitic, docosanoic, arachidonic, docosapentaenoic, arachidic, and docosahexaenoic acids represented the biosynthesis of unsaturated fatty acids pathway. A heat map revealed many correlations between gut microbial genera and distinct metabolites, as shown in Figure 7B, *Blautia, Fusicatenibacter* and *Bacteroides* was negatively correlated with Docosapentaenoic acid, Docosahexaenoic acid, Arachidonic acid, Palmitic acid, Arachidic acid, Stearic acid and Docosanoic Acid; *Prevotella* was positively correlated with above metabolites. The findings of fecal metabolomic and 16S analyses showed that docosahexaenoic acid (*r* = −0.811), palmitic acid (*r* = −0.818), stearic acid (*r* = −0.813) negatively correlated with the prevalence *Blautia* (Supplementary Table 7).

**Figure 8:**
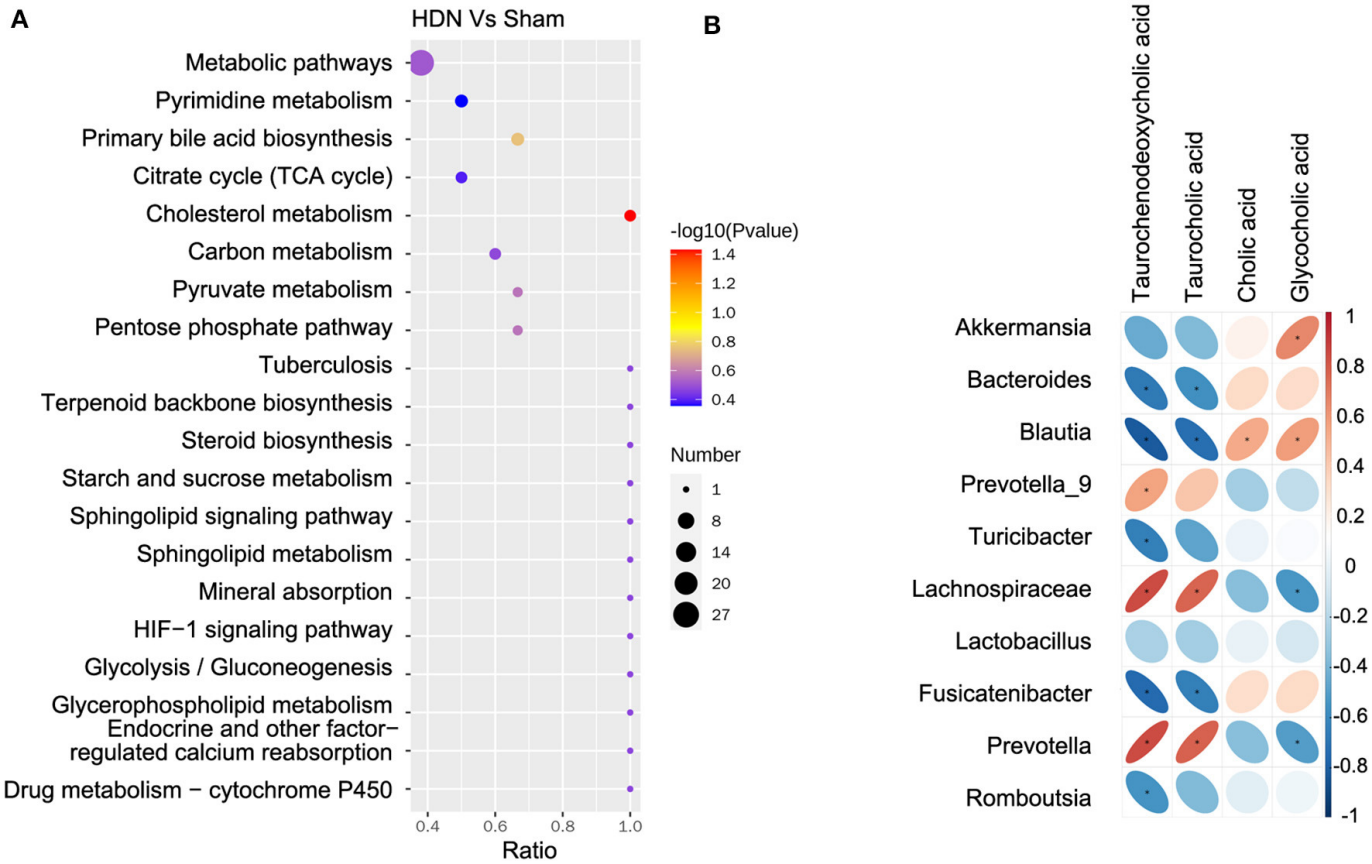
Correlations between microbiota and serum metabolites. **(A)** Serum metabolite changes in most relevant KEGG pathways. **(B)** Positive (red) and negative (blue) correlations between microbiota and serum metabolites. *P < 0.05. KEGG, Kyoto Encyclopedia of Genes and Genomes.

The KEGG analysis revealed 36 pathways that participated in serum metabolite synthesis (Supplementary Table 8). The cholesterol metabolism and primary bile acid biosynthesis pathways were significantly associated with serum metabolic alterations (Figure 8A). Among serum metabolites, taurochenodeoxycholic, taurocholic, and glycocholic acids represented the cholesterol metabolism pathway, and taurochenodeoxycholic, taurocholic, cholic, and glycocholic acids represented the primary bile acid biosynthesis pathway. Bile acids change the abundance of the microbiota by promoting the proliferation of bile-metabolizing bacteria and inhibiting the proliferation of bile-sensitive bacteria (Sayin et al., 2013). The microbiota can also regulate bile acid synthesis (Abenavoli et al., 2019). Therefore, we further analyzed relationships between the microbiota and bile acids. A heat map shows correlations between serum metabolites and gut microbial genera (Figure 8B). Taurochenodeoxycholic acid correlated negatively with the prevalence of *Blautia* (*r* = −0.815), and taurochenodeoxycholic acid correlated positively with Lachnospiraceae (*r* = 0.831) and *Prevotella* (*r* = 0.832) (Supplementary Table 9).

## 4 Discussion

In the current research, we compared changes in the composition and function of gut microbiota in HDN and sham rats using 16S rRNA gene sequencing, we further explored the relationship and function between microbiota and metabolites. Our results indicate that the metabolic spectrum, composition, and structure of the gut microbiota significantly differed between HDN and sham rats. The F/B ratio was notably higher in the HDN group than in the sham group. The abundance of *Proteobacteria* and *Verrucomicrobia* was substantially higher, whereas that of *Bacteroidetes* was significant lower in the HDN group than in the sham group. *Akkermansia, Bacteroides, Blautia, Turicibacter, Lactobacillus, Romboutsia, and Fusicatenibacter* were the most abundant, and *Prevotella, Lachnospiraceae_NK4A136_group*, and *Prevotella_9* were the least abundant in the HDN group. Further analysis with bile acid metabolites in serum showed that *Blautia* was negatively correlated with taurochenodeoxycholic acid, taurocholic acid, positively correlated with cholic acid and glycocholic acid in serum.

The F/B ratio is an indicator that typically reflects the dysbiosis of gut microbiota in various metabolic diseases, changes in this ratio can lead to a range of illnesses (Abenavoli et al., 2019). The F/B ratio was notably higher in the HDN group than in the sham group. *Bacteroidetes* was significant lower in the HDN group than in the sham group. Consistent with our results, the abundance of *Bacteroides* is decreased in patients with T2DM (Yamaguchi et al., 2016), and *Bacteroides* supplementation improves insulin resistance in diabetic mice (Yang et al., 2017). *Bacteroides* is a protective bacterium that plays a crucial role in glucose metabolism. *Bacteroidetes* produces short chain fatty acids, enhancing the function of the intestinal barrier (Chen et al., 2021). Short chain fatty acids, especially butyric acid, can activate GPR43, PPAR-γ, RASS system (Maslowski et al., 2009; Bolognini et al., 2016; Stino and Smith, 2017; Wysocki et al., 2017), lack of *Bacteroidetes* may promote the occurrence and development of HDN by activating GPR43, PPAR-γ, RASS system.

*Proteobacteria* are frontline responders that are sensitive to environmental factors such as diet (Shin et al., 2015). Excessive *Proteobacteria* growth is associated with inflammatory bowel disease and metabolic syndrome (Lavelle et al., 2015). Our findings on *Proteobacteria* was substantially higher are consistent with those of a meta-analysis of 578 patients with diabetic kidney disease (DKD) and 444 healthy persons (Wang et al., 2022), which revealed an enriched relative abundance of Proteobacteria in patients with DKD compared with healthy individuals.

We also found enriched abundance of *Akkermansia muciniphila* (phylum *Verrucomicrobia*) in the HDN group, *Akkermansia* aids in the development and preservation of the intestinal mucus layer, improves the functionality of the intestinal barrier, inhibits the proliferation of detrimental bacteria, and diminishes the concentration of intestinal endotoxins, thus protecting intestinal health (Zheng et al., 2023). *Akkermansia* reduces the risk of obesity, diabetes, enteritis, colon cancer and other diseases (Li et al., 2023; Zheng et al., 2023). However, the abundance of *A. muciniphila* is increased in experimental animals and humans with CKD (Lakshmanan et al., 2021), accompanied by an increase in indoxyl sulfate and p-cresyl sulfate (pCS). *A. muciniphila* in renal hypertension caused by CKD enhances the progression of renal hypertension by promoting inflammation (Lau et al., 2018). The inflammatory response is more active in renal hypertension (Rodriguez-Iturbe and Johnson, 2010), which might increase the abundance of *A. muciniphila*. Salt retention, endothelial dysfunction, volume overload, and abnormal hormone levels might results in HDN (Ku et al., 2019), these may increase mucus foraging in intestinal mucus layer. Besides that, in our HDN rat model, lack of fiber diet may lead to increased mucus foraging and increasing *Akkermansia* relative abundance.

*Blautia* was significantly more abundant in the HDN group, in direct proportion to various bile acids (BAs). *Blautia* is involved in converting primary to secondary Bas (Vojinovic et al., 2019). BAs exert toxic effects on the liver, kidney, intestine, stomach, and cardiovascular endothelial cells (Perez and Briz, 2009). BAs also regulating the activation of farnesoid X receptor (FXR), G protein-coupled 5 receptor, vitamin D receptor and pregnane X receptor (Wahlstrom et al., 2016). FXR, interacts with bile acids, haspotential protective effects on inflammatory and fibrotic damage in the CKD (Glastras et al., 2015). Researchers have used the FXR/TGR5 agonist int-767 to treat db/db mice, which can improve proteinuria, prevent podocyte damage, mesangial dilation, and renal tubulointerstitial fibrosis (Wang et al., 2018). In rats, taurochenodeoxycholic acid acts as an agonist of FXR in rats (Parks et al., 1999), cholic acid has an antagonistic effect on FXR, and the gut microbiota of mice benefits FXR signaling by reducing cholic acid (Sayin et al., 2013). In the present study, serum cholic acid was increased in HDN, which is positively correlated with the *Blautia* microbiota in feces, whereas taurochenodeoxycholic acid was decreased in HDN, negatively correlated with the *Blautia* microbiota in feces, suggesting that *Blautia* promotes HDN progression through the FXR signaling pathway regulated by bile acid metabolism. *Blautia* regulates the pathogenesis of HDN via the microorganism - gut - metabolite axis.

## 5 Conclusions

A disordered intestinal microbiota is closely associated with HDN. The F/B ratio was significantly increased in the HDN group, compared with the sham group. The most abundant bacteria in HDN were *Akkermansia, Bacteroides, Blautia, Turicibacter, Lactobacillus, Fusicatenibacter*, and *Romboutsia*; the least abundant flora were *Prevotella_9, Lachnospiraceae_NK4A136_group*, and *Prevotella*. Among them, *Blautia* is an important inducer of HDN. *Blautia* was negatively correlated with taurochenodeoxycholic acid, taurocholic acid, positively correlated with cholic acid, and glycocholic acid in serum, which might regulate pathogenesis through the microorganism – gut – metabolite axis. However, the present study has a limitation. It is the relatively small sample size of the study. Our finding of a correlation between intestinal microbiota and metabolites in HDN might pave the way toward therapy targeting the intestinal microbiota of patients with HDN.

## Data availability statement

The data presented in the study are deposited in the figshare repository, accession number is 10.6084/m9.figshare.25623453.

## Ethics statement

The animal study was approved by the Institutional Animal Care and Use Committee of Shengjing Hospital of China Medical University (Shenyang, China). The study was conducted in accordance with the local legislation and institutional requirements.

## Author contributions

DP: Writing – review & editing, Writing – original draft, Supervision, Funding acquisition, Conceptualization. JL: Writing – review & editing, Writing – original draft, Methodology, Funding acquisition, Formal analysis, Conceptualization. XG: Writing – review & editing, Writing – original draft, Formal analysis, Data curation. CZ: Data curation, Supervision, Writing – original draft, Writing – review & editing. TY: Writing – review & editing, Writing – original draft, Methodology, Investigation, Formal analysis.
