# Supplementary material for: A multi-omics approach to investigate characteristics of gut microbiota and metabolites in hypertension and diabetic nephropathy SPF rat models
Source: Front Microbiol. 2024 Apr 29;15:1356176. doi: 10.3389/fmicb.2024.1356176 (PMC11089221; doi:10.3389/fmicb.2024.1356176)
Supplement: Supplementary file 1 [file Table_1.DOCX]

# **Supplementary Figure Captions**

**Supplementary Figure 1**.

**(A)** Sankey map showed relative abundance of the screened top5 species at the Phylum level.

**(B,C)** Rarefaction curve evaluating the relative bacterial richness to determine whether further sequencing would identify additional OTUs. Sham group(blue); HDN group(red).**(D)** Rank Abundance.**(E)** Rank Abundance by group analysis.**(F)** Species accumulation box plots.

**Supplementary Figure 2.** Alterations in the composition of fecal microflora associated

with HDN. **(A)** Welch’s t-test results for evaluating the relative abundance of significantly

different microbiota at the phylum level. Sham (yellow) and HDN (blue) groups for bars and dots. **(B)** Welch’s t-test results for evaluating the relative abundance of significantly

different microbiota at the genus level. Sham (yellow) and HDN (blue) groups for bars and dots.
