# Supplementary material for: A multi-omics approach to investigate characteristics of gut microbiota and metabolites in hypertension and diabetic nephropathy SPF rat models
Source: Front Microbiol. 2024 Apr 29;15:1356176. doi: 10.3389/fmicb.2024.1356176 (PMC11089221; doi:10.3389/fmicb.2024.1356176)
Supplement: Supplementary file 2 [file Table_2.DOCX]

**Supplementary table captions**

**Supplementary Table 1.** The alpha diversity analysis indexes (shannon, simpson, chao1, ACE, goods_coverage, PD_whole_tree) of different samples at 97% consistency threshold are statistically analyzed.

**Supplementary Table 2.** At phylum level analysis shows significantly different between the Sham and HDN groups.

**Supplementary Table 3.** At genus level, 114 genera were significantly different between the Sham and HDN groups.

**Supplementary Table 4.** Compared with the Sham group, the down-regulated fecal metabolites in the HDN group.

**Supplementary Table 5.** Compared with the Sham group, the down-regulated serum metabolites in the HDN group.

**Supplementary Table 6.** KEGG analysis revealed that synthesis of fecal metabolites was regulated by 69 different metabolic pathways.

**Supplementary Table 7.**Multiple correlations were observed between the gut microbiota at the genus level and fecal metabolites.

**Supplementary Table 8.** KEGG analysis revealed that synthesis of serum metabolites was regulated by 36 different metabolic pathways.

**Supplementary Table 9.**Multiple correlations were observed between the gut microbiota at the genus level and serum metabolites.
