# Supplementary figures and images for: A multi-omics approach to investigate characteristics of gut microbiota and metabolites in hypertension and diabetic nephropathy SPF rat models
Source: Front Microbiol. 2024 Apr 29;15:1356176. doi: 10.3389/fmicb.2024.1356176 (PMC11089221; doi:10.3389/fmicb.2024.1356176)

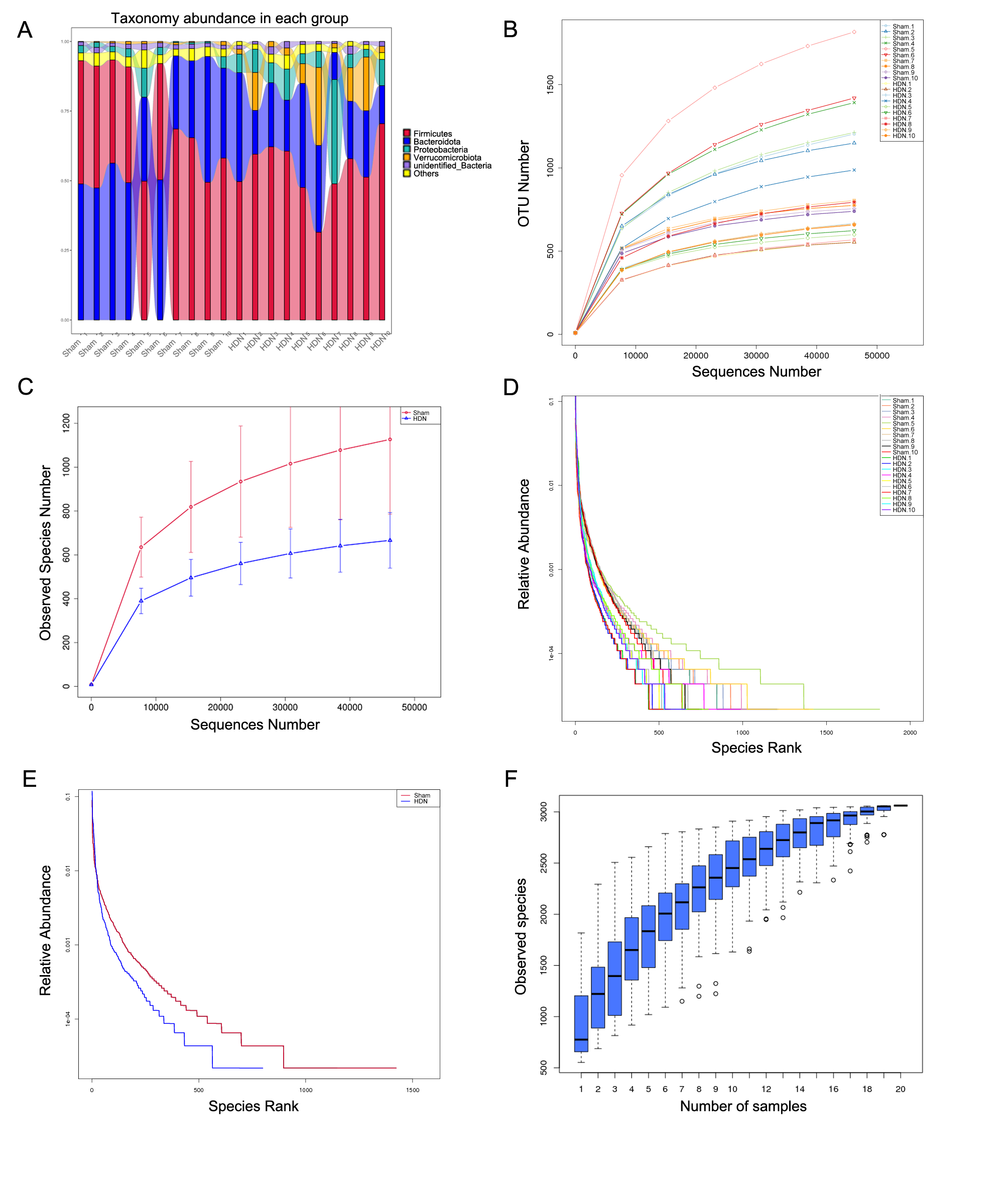

Supplement: Supplementary file 5 [file Image_1.TIF]

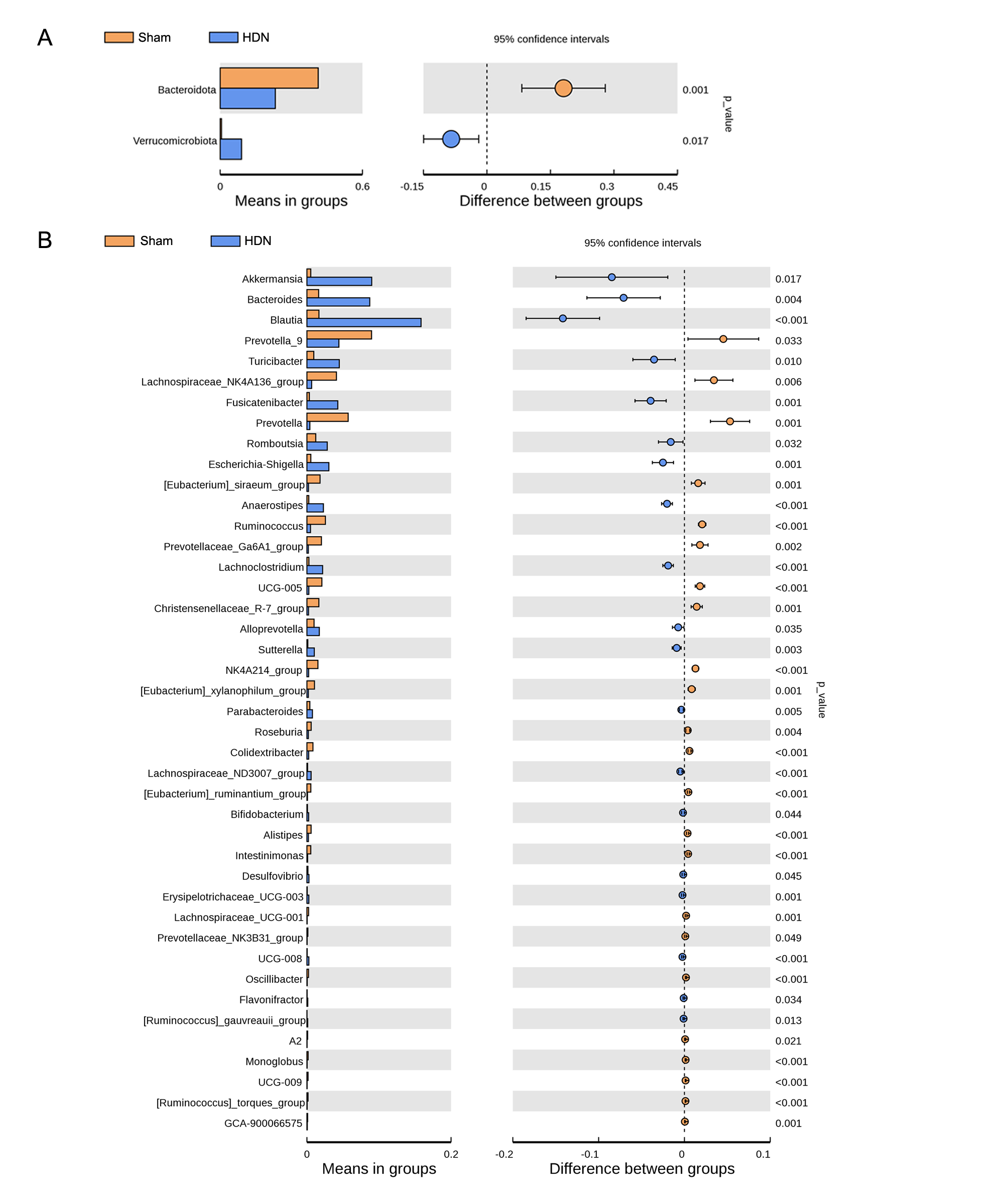

Supplement: Supplementary file 6 [file Image_2.TIF]
